# Retention of Memory through Metamorphosis: Can a Moth Remember What It Learned As a Caterpillar?

**DOI:** 10.1371/journal.pone.0001736

**Published:** 2008-03-05

**Authors:** Douglas J. Blackiston, Elena Silva Casey, Martha R. Weiss

**Affiliations:** Department of Biology, Georgetown University, Washington, D. C., United States of America; University of Edinburgh, United Kingdom

## Abstract

Insects that undergo complete metamorphosis experience enormous changes in both morphology and lifestyle. The current study examines whether larval experience can persist through pupation into adulthood in Lepidoptera, and assesses two possible mechanisms that could underlie such behavior: exposure of emerging adults to chemicals from the larval environment, or associative learning transferred to adulthood via maintenance of intact synaptic connections. Fifth instar *Manduca sexta* caterpillars received an electrical shock associatively paired with a specific odor in order to create a conditioned odor aversion, and were assayed for learning in a Y choice apparatus as larvae and again as adult moths. We show that larvae learned to avoid the training odor, and that this aversion was still present in the adults. The adult aversion did not result from carryover of chemicals from the larval environment, as neither applying odorants to naïve pupae nor washing the pupae of trained caterpillars resulted in a change in behavior. In addition, we report that larvae trained at third instar still showed odor aversion after two molts, as fifth instars, but did not avoid the odor as adults, consistent with the idea that post-metamorphic recall involves regions of the brain that are not produced until later in larval development. The present study, the first to demonstrate conclusively that associative memory survives metamorphosis in Lepidoptera, provokes intriguing new questions about the organization and persistence of the central nervous system during metamorphosis. Our results have both ecological and evolutionary implications, as retention of memory through metamorphosis could influence host choice by polyphagous insects, shape habitat selection, and lead to eventual sympatric speciation.

## Introduction

Holometabolous insects undergo radical changes not just in body form, but also in life style, diet, and dependence on particular sensory modalities as they proceed through complete metamorphosis. Indeed, it is hard to believe that a cryptic caterpillar chewing on a leaf, or a maggot wriggling in decaying flesh, is in fact the same animal as the colorful butterfly or noisy blowfly emerging from the transitional pupal stage. In light of these radical changes, might it be possible for learned associations formed at the larval stage to be accessible to the adult? This intriguing and controversial idea challenges our understanding of neuronal fate during metamorphosis in holometabolous insects. If associative behavior is indeed retained across the pupal stage, might it result from the persistence of larval neurons through metamorphosis and their subsequent integration into the adult nervous system [1], [2]? Or is the reorganization of the insect nervous system during metamorphosis so dramatic that it would preclude the persistence of neuronally-based chemosensory memory [3]?

Studies in a handful of taxa, including hymenopterans, dipterans, coleopterans, and lepidopterans, have suggested that larval experience can indeed affect adult behavior. Adult responses to larval experience have been reported in various contexts, including location and acceptance of food sources or oviposition substrates, as well as recognition of nest-mates [1], [2], [4]–[7]. However, the mechanisms underlying this behavioral carryover remain controversial. In some cases, changes in adult behavior have been shown to result not from memory of larval experience, but rather from exposure of the newly pupated adults to chemicals carried over from the larval environment [5], [8], [9]. In other instances, however, the behavior seems to reflect actual persistence of larval associative learning into adulthood.

A connection between larval and adult behavior can develop if emerging adults are exposed to odors carried over from the larval environment, a phenomenon termed ‘chemical legacy’ [10]. Support for this idea comes from studies in which pupae are cleaned or physically separated from olfactory cues associated with the larval environment, thereby eliminating the opportunity for habituation or sensitization of the emerging adults [5], [8]. For example, washing the pupal cases of *Drosophila* that had been reared as larvae on menthol-scented diet eliminated an adult attraction to the odor, whereas application of menthol to the pupal cases of larvae naive to that odor resulted in an increased attraction to menthol in the emergent adults [9]. However, several investigations have explicitly prevented exposure of emergent adults to larval environmental stimuli [2], [4], [6], [11], and have nonetheless observed an effect of larval experience.

In the cases for which chemical legacy has been ruled out, it has been postulated that the connection between larval and adult experience could result from the survival of larval neurons during metamorphosis, enabling persistence in the adult brain of memories formed during the larval stage [2], [12]. If olfactory memories are retained across metamorphosis, they are likely to be located in the mushroom bodies (MB), paired structures in the larval and adult insect brain that receive input from the antennal lobes [13]–[15]. The fate of the MB cells during the transition from larva to adult is poorly understood. In *Drosophila*, the only holometabolous insect for which individual MB neurons have been tracked through metamorphosis, a subset of the larval neurons maintain intact projections into adulthood [12], while many of the other MB neurons are pruned to the main process prior to production of adult-specific projections [12], [16]. Thus it is possible that synaptic connections may persist through metamorphosis and carry memory from larva to adult, although this hypothesis has yet to be tested.

If synaptic connections do indeed persist through metamorphosis, the carryover of larval memory into adulthood might depend on the timeframe of larval experience. In *Drosophila*, those MB neurons that are pruned prior to pupation form early in larval development, whereas those that persist through metamorphosis are formed later [12]. Thus memory of later larval experience may persist into adulthood, while memory of early experience may not.

Among holometabolous insects, retention of memory through metamorphosis may be of particular ecological importance for lepidopterans, as carryover of larval experience into adulthood could enable polyphagous species to preferentially oviposit on their own larval host plant (Hopkins' host-selection principle (HHSP)) [3], [5], [17]–[20]. Furthermore, learning is well documented in both larval [21]–[23] and adult lepidopterans [24]–[28]. Several studies have documented an effect of caterpillar experience on adult oviposition behavior [19], [29], [30], but have attributed the effect to chemical legacy, or have not been able to rule out this possibility, and thus have not demonstrated persistence of a learned association across metamorphosis.

Here we assess whether or not true associative memory persists through metamorphosis in the tobacco hornworm, *Manduca sexta* (Lepidoptera: Sphingidae). We ask: (1) Do larvae learn aversive cues? (2) Does aversive behavior persist across larval molts? (3) Does aversive behavior persist through metamorphosis? (4) Does persistence of memory into adulthood depend on the timing of larval experience? (5) Does exposure to chemicals from the larval environment at the time of eclosion influence adult behavior?

## Results

### Do larvae learn aversive cues?

**Figure 1:**
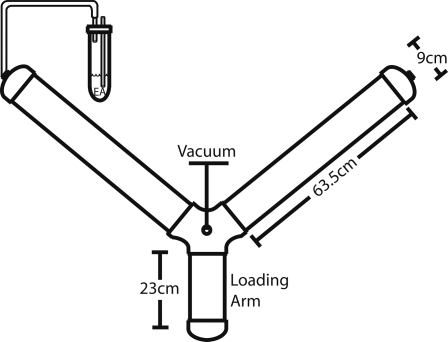
Diagram of Y choice apparatus used for larval and adult testing. Individual *M. sexta* were placed into a short “loading arm” attached to a 10cm diameter central chamber to which a vacuum was applied. Air was bubbled through 20 ml of EA and pulled through one of the two side arms, while ambient air was pulled through the other. Larvae and adults were allowed to move freely within the apparatus for ten minutes, at which time their position was scored as either the EA arm, ambient air arm, or no choice (defined as the loading arm or any portion of the central chamber).

**Figure 2:**
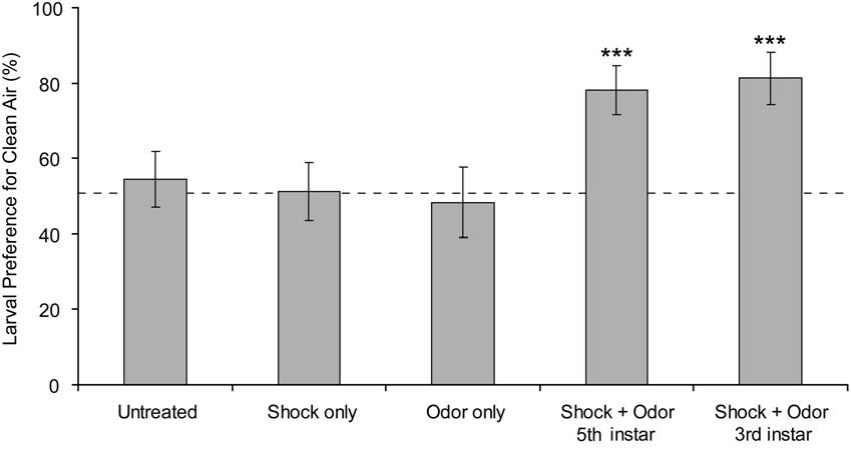
Larvae conditioned with forward-paired shock+odor avoid EA at fifth instar. Proportion of *M. sexta* larvae choosing ambient air rather than EA in the Y choice apparatus after receiving one of five treatments: no exposure to odor or electric shock (N = 46), shock only (N = 43), odor only (N = 29), the forward pairing of shock+odor at fifth instar (N = 41), or forward pairing of shock+odor at third instar (N = 32). Only larvae conditioned with shock+odor demonstrate a significant aversion to EA as larvae, and larvae trained at third instar recall the aversion at fifth instar, indicating retention of memory across molts. *** (p<0.001) indicates values that differ significantly from random choice (dashed horizontal line) by a two-tailed binomial calculation. Values are means±SD.

To investigate learning in *M. sexta* larvae, we used classical conditioning to train caterpillars to avoid the odor of ethyl acetate (EA) by pairing it with a mild electric shock. When offered the choice of ambient air or EA-scented air in a Y choice apparatus (Figure 1), naive fifth instar caterpillars showed neither attraction nor aversion to the odor of EA (Figure 2, binomial calculation, N = 46, p = 0.32). Similarly, larvae exposed to shock alone showed no attraction or aversion to EA (N = 43, p = 1.0). The same lack of discrimination was seen in larvae exposed to EA in the absence of shock, suggesting that neither habituation nor sensitization occurred with repeated exposure to the odor (N = 29, p = 1.0). However, the forward pairing of EA with electric shock (odor prior to shock) through eight training bouts produced a significant aversion in fifth instar larvae (N = 41, p<0.001), with 78% of caterpillars choosing ambient air over EA. Backward pairing of odor and shock (odor following shock) produced no change in behavior relative to control caterpillars (N = 22, p = 0.52, data not shown).

### Does aversive behavior persist across larval molts?

Pairing of EA odor with electric shock during early third instar produced a significant aversion to EA in larvae tested 10 to 14 days later as late fifth instars (Figure 2, binomial calculation, N = 32, p<0.001), with 81% of larvae preferring ambient air over EA. This level of response was similar in magnitude to that of larvae trained and tested at fifth instar (chi-square test for equality of distributions, chi-square = 0.1128, DF = 1, p = 0.74), indicating that the time interval between third and fifth instars did not result in a diminution of response, and also that larvae trained at both stages demonstrated similar levels of aversion just prior to pupation.

### Does aversive behavior persist through metamorphosis?

**Figure 3:**
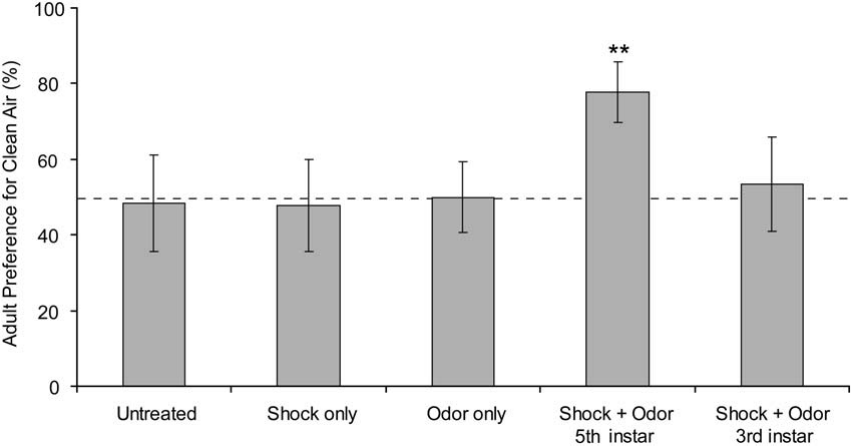
Larvae conditioned with forward-paired shock+odor at fifth instar retain odor avoidance as adults. Proportion of adult *M. sexta* choosing ambient air rather than EA in the Y choice apparatus after receiving one of five treatments: no exposure to odor or electric shock (N = 31), shock only (N = 23), odor only (N = 28), the forward pairing of shock+odor at fifth instar (N = 27), or forward pairing of shock+odor at third instar (N = 15). Only individuals that received the shock+odor pairing as fifth instar caterpillars maintained odor avoidance as adults. While individuals trained at third instar demonstrated odor aversion at fifth insta (Fig 2.), the behavior was lost during pupation. ** (p<0.01) indicates the value that differs significantly from random choice (dashed horizontal line) by a two-tailed binomial calculation. Values are means±SD.

To determine whether adult *M. sexta* that had learned to avoid the odor of EA as larvae still exhibited odor avoidance behavior following metamorphosis, individuals from each fifth instar larval treatment were retested for odor preference as adults, approximately 28 to 35 days after larval conditioning, using the same Y choice apparatus. We saw neither attraction nor aversion to EA in moths that as larvae were not exposed to EA (binomial calculation, N = 31, p = 1.0), were exposed to EA alone (N = 23, p = 1.0), or were shocked in the absence of EA (N = 28, p = 1.0 (Figure 3). In marked contrast, adults emerging from the fifth instar forward-paired shock+odor treatment showed a level of aversion to EA similar to that shown by the larvae, with 77% of adults choosing ambient air (N = 27, p = 0.005). Thus, aversive behavior acquired during the larval stage was retained through metamorphosis. To determine whether larval choice predicted adult choice, for each treatment we calculated a ‘constancy’ score, defined as the proportion of individuals choosing ambient air as larvae that also chose ambient air as adults. Constancy measures of adults developing from naïve larvae, from larvae exposed to odor only, and from larvae exposed to shock only, were not significantly different from 50%, suggesting random choice at the adult stage. However, adults from the forward-paired shock+odor treatment demonstrated 80% constancy, indicating that the majority of individuals choosing ambient air as adults had also chosen ambient air as larvae (binomial calculation, N = 15, p = 0.035).

### Does persistence of memory into adulthood depend on the timing of larval experience?

In contrast to larvae trained to forward-paired shock+odor at fifth instar, which maintained odor aversion as adults, larvae trained to avoid EA as early third instars did not avoid EA as adults (Figure 3, binomial calculation, N = 15, p = 0.5).

### Does exposure to chemicals from the larval environment at the time of eclosion influence adult behavior?

**Figure 4:**
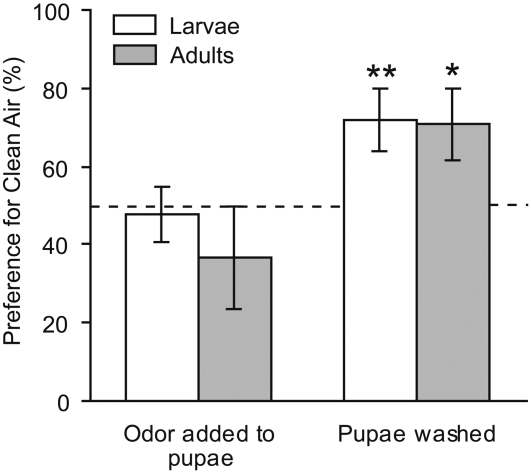
Aversion to EA in adults is not due to exposure to odors from the larval environment. Proportions of untreated *M. sexta* larvae (light bars) and adults (dark bars) that had an EA-impregnated gel added to their pupal case (N = 40 adults, 30 larvae), and of shock+odor conditioned larvae whose pupal cases were washed (N = 39 adults, 31 larvae), that chose ambient air over EA in the Y choice apparatus. * (p<0.05) or ** (p<0.01) indicates values that differ significantly from random choice (dashed horizontal line) by a two-tailed binomial calculation. Values are means±SD.

To determine whether the presence of chemical residues on pupal cases might account for the observed changes in adult behavior, we applied EA odors to the pupae of naive larvae and removed any residual EA odors from EA-conditioned larvae by washing their pupae. Application of EA-impregnated gel to the pupal cases of untreated larvae did not result in odor aversion in adult moths (Figure 4, binomial calculation, N = 30, p = 0.20); nor did washing the pupal cases of larvae conditioned with forward-paired shock+odor result in a loss of odor aversion in adults (N = 31, p = 0.438). In addition, control pupae were washed and demonstrated no change in behavior as adults compared to unwashed larvae, indicating no effect of mechanical stimulation during the pupal stage (N = 25, p = 0.423). Thus, chemical legacy or contamination cannot account for the persistence of EA avoidance in adult moths.

## Discussion

We have demonstrated that *M. sexta* larvae can learn to associate odor cues with an aversive stimulus, and that this memory persists undiminished across two larval molts, as well as into adulthood. The behavior represents true associative learning, not chemical legacy, and, as far as we know, provides the first definitive demonstration that associative memory survives metamorphosis in Lepidoptera. Furthermore, the results from our differential timing of larval training are consistent with the idea that retention of memory could be due to the persistence into adulthood of intact larval synaptic connections.

Our results support those of a handful of other studies that show learning within a larval instar in Lepidoptera [23], [31], [32]. Only the forward temporal pairing of training odor with electric shock generated aversive behaviors in larvae. Backward pairing of shock and EA did not result in an aversive response to EA in larvae; nor did exposure to EA alone or shock alone cause larvae to avoid EA, ruling out the possibility that the behavior was a result of sensitization or habituation to either stimulus. Interestingly, larval food aversion learning has not been observed in *Manduca sexta* [32], nor in several other lepidopteran taxa [33], [34] despite the extreme negative consequences of ingestion of the noxious or toxic food.

Like fifth instar larvae, third instar *M. sexta* caterpillars could be conditioned to avoid EA, and they recalled this information 10–14 days later, after two molts, as late fifth instars. We know of only one other study that demonstrates retention of larval memory across a molt in Lepidoptera: neonate larvae of the codling moth *Cydia pomonella* (Tortricidae) recall a learned aversion to noxious food across four days during the transition from first to second instar [23].

Over the past century, a number of investigators have experimentally evaluated the effect of larval experience on adult behavior in beetles, moths, and butterflies [29], [30], [35], [36]. Some of these studies have sought to assess the validity of Hopkins' Host Selection Principle [3], [20] whereas others have explicitly examined the persistence of associative memory across metamorphosis. Evidence for HHSP, whether based on chemical legacy or retention of memory across metamorphosis, is equivocal. In some lepidopteran species, adults show an increased tendency to oviposit on their larval host plant [19], [29], [30], while in others larval feeding experience has no effect on adult host plant preference [35], [37], [38]. Of those studies that do show an effect of larval experience on adult behavior, none have ruled out the possibility of chemical legacy. For example, Chow et al. [30] demonstrated that oviposition deterrence in the presence of a novel chemical was markedly reduced following larval consumption of the chemical. Though pupae were removed from the larval environment and rinsed prior to the adult trials, residues of the non-water-soluble chemical may still have been present in the insect haemolymph or outside the pupal case.

In our experimental design, we attempted to eliminate the possibility of chemical carryover from the larval environment by using an electric shock rather than an aversive ingested chemical as the unconditioned stimulus, and by using ephemeral exposure to a gaseous compound, EA, as the conditioned stimulus. To further ensure that chemical carryover was not a factor, we washed pupae that had experienced forward-paired shock+odor as larvae, and applied EA to pupae that developed from naïve larvae, and in neither case did our results change relative to the experimental treatments. Thus, we are confident that the observed changes in adult behavior reflect larval experience, rather than exposure of emergent adults to cues from the larval environment.

Our results demonstrate a clear effect of larval experience on adult behavior. Only the pairing of training odor with electric shock generated aversive behaviors in larvae, and this aversion was retained in the adult moths. Furthermore, our constancy calculations demonstrate that the majority of individuals in the forward-paired shock+odor treatment made the same choice as larvae and as adults, indicating that individual preferences were maintained.

Studies of a handful of other holometabolous taxa, including beetles [4], fruit flies [2], ants [7] and parasitic wasps [6], [11], have convincingly demonstrated an effect of larval experience on adult behavior that was not due to exposure of emergent adults to residual chemicals, and several have suggested, but have not explored, a neural basis for their findings. What mechanisms could account for the carryover of larval experience into adulthood in our system? Manipulation of the timing of larval conditioning may provide insight into the basis of memory retention, as regions of the MBs develop at different times, and have different fates; that is, some lobes are retained intact through metamorphosis while others are not. Our results are consistent with, but do not provide conclusive support for the survival of synaptic connections within the larval brain across metamorphosis, enabling persistence in the adult brain of memories formed during the larval stage.

We found that adults that developed from larvae trained at fifth instar recalled their larval experience, whereas those that were trained at third instar did not. In *Drosophila*, the only holometabolous insect for which individual MB neurons have been tracked through metamorphosis, development of the tri-lobed MB occurs in a sequential fashion, with the γ lobe forming embryonically, the α′/β′ lobe developing just prior to pupation, during mid-third instar, and neurogenesis of the α/β lobe initiating at the onset of pupation [12], [16]. During pupation, γ lobe neurons are pruned to the main process prior to production of adult-specific projections, while α′/β′ neurons maintain intact projections throughout metamorphosis [12]. Since *M. sexta* progress through five instars prior to pupation while *Drosophila* progress through only three, it is likely that third instar training in *M. sexta* occurs before α′/β′ neurogenesis. If *M. sexta* MB development is analogous to that of *Drosophila*, then our findings are consistent with the idea that the memory resulting from third instar training depends upon the embryonically-formed γ lobe, which is intact at fifth instar and so could enable recall at that stage, but is lost in adults subsequent to γ lobe pruning during pupation. The memory resulting from fifth instar training, however, could be retained in the later-forming α'/β' lobe, which remains intact throughout pupation and could therefore allow recall at the adult stage. As such, it would be interesting to examine the effects of α′/β′ ablation on adult memory of larvae trained at late instars.

Many studies of insect learning use appetitive as opposed to aversive training to mimic positive feeding experiences that occur in nature. Honeybees, butterflies and moths, for example, have been shown to associate both colors and odors with food rewards [25], [26], [39]–[44]. However, insects also learn aversive cues in a variety of ecological contexts. For example, mantids rapidly learn to avoid noxious and aposematically colored milkweed bugs [45] and *Manduca sexta* larvae become sensitized to repeated pinching (analogous to bird attacks), showing increased defensive behavior in response to recurring assailment [46]. Thus, although the current study uses an artificial electrical shock as the aversive stimulus, this type of conditioning is consistent with aversive experiences in nature.

Duration of associative memory in insects varies considerably, from minutes to months, depending on identity, age, and gender of test organism, strength of rewarding or aversive stimulus, number of training repetitions, and assay type [47]. These variables notwithstanding, memory of aversive conditioning often lasts longer than that of appetitive conditioning [2], [23], [47]–[49]. In the current study, avoidance of EA by *M. sexta* subjected to forward-paired shock+odor was almost identical before and after the 4–5 week pupal period (78% of larvae and 77% of adults avoided EA), demonstrating a long-lasting and stable memory. A similarly long-lasting aversive memory is seen in the hemimetabolous cricket, *Gryllus bimaculatus*, which retained an association between an odor and salt water for up to 10 weeks [48].

The present study has both ecological and evolutionary implications. as retention of memory through metamorphosis could influence host choice by polyphagous insects, and could further lead to the formation of host races or even to eventual sympatric speciation [17], [50], [51]. While some studies of this phenomenon suggest chemical legacy as the process by which HHSP occurs, our data also implicate retention of memory, although both could lead to the same result [4]. In addition, the mechanism for HHSP could vary between taxa, as is observed in lepidopteran host plant induction [52].

Carryover of larval experience into adulthood could have important consequences not just for insects in nature, but also for laboratories studying adult insects. Larval “chemical legacies” have already been shown to generate spontaneous odor attraction in adults [5], [8]–[10]. Furthermore, evidence suggests that larval artificial diets can impact aspects of adult physiology, such as color vision [53]. These observations, in conjunction with the survival of memory across metamorphosis demonstrated in the present study, argue for the standardization of rearing conditions and protocols between labs. Variation in factors as seemingly irrelevant as larval environment, diet, or cage color could lead to unexpected effects on adult behavior, which could then contribute to significant variation in observations between labs, or the inability to replicate results if animals are obtained from different rearing facilities.

Our behavioral results are exciting not only because they provoke new avenues of research into the fate of sensory neurons during pupation, but also because they challenge a broadly-held popular view of lepidopteran metamorphosis: that the caterpillar is essentially broken down entirely, and its components reorganized into a butterfly or moth. Further studies of neuronal fate in holometablous organisms will yield greater insight into the process of complete metamorphosis and move us closer to an integrated understanding of organisms, providing links between complex cognitive behaviors and the molecules and developmental processes that give rise to them.

## Materials and Methods

### Study Taxa

*M. sexta* larvae were obtained from the North Carolina State University insectary and housed in 10.5×14×28 cm plastic rearing containers. Caterpillars for third instar experiments were reared from eggs, while those used for fifth instar experiments included larvae obtained at fourth instar as well as those reared from eggs. Up to 12 individuals were raised in a single container, under 14 hr: 10 hr light/dark cycle at 24±2°C, 65% relative humidity. All larvae were reared on artificial diet containing 15 gm/liter agar, 100 gm/liter wheat germ, 45 gm/liter vitamin-free casein, 40 gm/liter sucrose, 30 gm/liter yeast, 15 gm/liter CaCl2, 1.5 gm/liter sorbic acid, 1 gm/liter methyl paraben, and 0.5 gm/liter cholesterol until pupation (recipe from North Carolina State University insectary). Late fifth instar larvae were moved to clean plastic containers and allowed to pupate beneath a layer of tissue paper under 24 hr darkness and 65% relative humidity. Adults were marked daily to track age, and were tested 3 days post-eclosion, as anecdotal communications report adult moths prior to this time display abnormal behavior. At least three separate cohorts of *M. sexta* were used for each treatment.

### Larval conditioning

Two to three days after molting to fifth instar, up to 10 *M. sexta* larvae were placed in a 25×14×10 cm clear plastic container, the bottom of which was lined with an 8 mm thick 2% agarose gel made conductive with 2 mM lithium chloride. Copper electrodes were embedded in the gel at opposite corners of the chamber. A vacuum was applied to one end of the closed chamber, and air was pulled unidirectionally through the apparatus via an opposite side arm containing 20 ml of EA in a 50 ml falcon tube. Larvae were subjected to 10 seconds of the odor alone, followed by 10 seconds of odor plus a continuous electrical shock. 90v (AC) was passed through the gel; individual caterpillars received a 16–18v (AC) shock depending on their size and position. Ten seconds after completion of conditioning, larvae were returned to their rearing boxes in order to minimize exposure to residual odors. This procedure was repeated seven additional times, once every hour, for a total of eight training sessions in a single day. Hour-long resting periods between each training bout allowed larvae to revert to normal feeding behavior. The number of training sessions necessary for learning was determined in a pilot study, the voltage was adapted from work done on *Drosophila,* and we chose ethyl acetate as a conditioning odor based on its high volatility and use in other behavioral studies [2]. In addition to the forward-paired shock+odor treatment, the following four controls were performed: 10 seconds of ambient air followed by 10 seconds of shock and ambient air (shock only), 20 seconds of EA odor alone (odor only), 20 seconds of ambient air (untreated), and 10 seconds of shock alone followed by a 10 second resting period with ambient air before 10 seconds of odor (backward pairing).

### Larval testing

The day after training, fifth instar larvae were tested for conditioned odor avoidance in a plexiglas Y choice apparatus (Figure 1). Individual larvae were placed in a short 23×9×9 cm “loading arm” attached to a 10 cm diameter central chamber to which a vacuum was applied. Air was bubbled through 20 ml of EA and pulled into one of the two 63.5×9×9 cm side arms, and ambient air was pulled through the other. An adaptor brought the vacuum in the central chamber roughly half way down from the top of the tube, in order to ensure that odor plumes were situated at a vertical level detectable to *M. sexta*. Testing was carried out in total darkness to eliminate lighting differences between the arms, and larvae were allowed to move freely within the Y tube for ten minutes. Pilot studies established ten minutes to be long enough for *M. sexta* to make choices but short enough to avoid random movement due to hunger. Upon completion of the 10 minute testing period, larval location was scored by a blind observer as either the EA arm, ambient air arm, or no choice (defined as the loading arm or any portion of the central chamber); larvae were then placed in separate rearing boxes based upon their choice of arm, and remained there until pupation. The entire apparatus was rotated 180° between batches of individuals to minimize any directional biases, and the central chamber and arms were cleaned after every three trials.

### Third instar training

The day following the molt to third instar, larvae were conditioned to avoid EA using the same procedure designed for fifth instars. Larvae were tested for odor avoidance at fifth instar in the Y maze as previously described; they were not tested as third instars so as to minimize exposure to EA without the unconditioned stimulus (electric shock), and due to logistical problems with the small size of the caterpillars in an apparatus developed for larger individuals. Upon completion of larval testing, individuals were allowed to pupate, and were then tested as adults for memory retention as above.

### Adult testing

Three days post-eclosion, individual adult *M. sexta* of each treatment were placed in the loading arm of the Y choice apparatus described above. Again, ambient air or EA-impregnated air was pulled through separate arms of the apparatus to the central chamber, and adults were allowed to walk between the arms for 10 minutes. The entire test was conducted in the dark. Upon completion of the 10 minute testing period, the location of the adult was scored by a blind observer as either the EA arm, ambient air arm, or no choice. The entire apparatus was rotated 180° between batches of individuals.

### Contamination controls

To add EA contamination, we applied 10 µl of a 2% agarose gel impregnated with a 10% ethyl acetate solution to the dorsal thoratic region of untreated control pupae. Gel was used to ensure that no liquid EA entered the spiracles, and to decrease the volatilization rate of the odor. Agarose was refreshed twice weekly, starting three weeks after pupation for a total of four to six applications. To remove any possible external contamination, the pupal cases of trained larvae (shock+odor) were washed thoroughly with a soft brush and distilled water one and three weeks after pupation (methods from Barron and Corbet, 1999). Three days post-eclosion, individuals from both treatments were tested for odor aversion in the Y choice apparatus, described above. In addition, untreated control larvae were washed to determine if mechanical stimulation at the pupal stage resulted in in a change of adult behavior.

### Statistics

All statistical analyses were performed using SPSS v. 14.0 (SPSS Inc., Chicago, IL). For each treatment the proportion of larvae or adults choosing the ambient air arm vs the EA arm was compared to an expected value of 50 percent with a two-tailed binomial calculation. Two-tailed tests were employed in order to detect a possible attraction to EA in contamination experiments. Constancy, the percentage of adults that made the same choice in the Y chamber as they did as larvae, was examined with a two- tailed binomial calculation with the expectation that fifty percent of individuals choosing no odor as adults made the same choice as larvae. Learning between larvae trained at third and fifth instar was compared with a chi-squared test of equality of distributions. Power tests were conducted with α = 0.05 and β = 0.8 to determine minimal sample sizes for all behavioral assays.
